# Copper-enriched hydroxyapatite coatings obtained by high-velocity suspension flame spraying. Effect of various gas parameters on biocompatibility

**DOI:** 10.1007/s10856-024-06846-3

**Published:** 2024-11-30

**Authors:** Long-Quan R. V. Le, M. Carolina Lanzino, Matthias Blum, Anika Höppel, Ali Al-Ahmad, Andreas Killinger, Rainer Gadow, Wolfgang Rheinheimer, Michael Seidenstuecker

**Affiliations:** 1 G.E.R.N. Center of Tissue Replacement, Regeneration & Neogenesis, Department of Orthopedics and Trauma Surgery, Faculty of Medicine, Albert-Ludwigs-University of Freiburg, Hugstetter Straße 55, 79106 Freiburg, Germany; 2 Institute for Manufacturing Technologies of Ceramic Components and Composites, University of Stuttgart, 70569 Stuttgart, Germany; 3 Department Tissue Engineering and Regenerative Medicine (TERM), University Hospital Würzburg, 97070 Würzburg, Germany; 4 Department of Operative Dentistry and Periodontology, Center for Dental Medicine, Faculty of Medicine, Albert-Ludwigs-University of Freiburg, 79106 Freiburg, Germany

## Abstract

Hydroxyapatite (HAp)-coated bone implants are frequently used for orthopaedic or dental implants since they offer high biocompatibility and osteoconductivity. Yet, problems such as infections, e.g. periprosthetic joint infections, occur when implanting foreign material into the body. In this study, HAp coatings were produced via high-velocity suspension flame spraying (HVSFS). This method allows for the production of thin coatings. We investigated the effects of different gas parameters on the coating properties and on the biocompatibility, which was tested on the human osteosarcoma cell line MG63. Furthermore, Copper (Cu) was added to achieve antibacterial properties which were evaluated against standard microorganisms using the airborne assay. Three gas parameter groups (low, medium, and high) with different Cu additions (0 wt.%, 1 wt.% and 1.5 wt.%) were evaluated. Our findings show that porosity as well as hardness can be controlled through gas parameters. Furthermore, we showed that it is possible to add Cu through external injection. The Cu content in the coating as well as the release varies with different gas parameters. Both antibacterial efficacy as well as biocompatibility are affected by the Cu content. We could significantly reduce the amount of colony-forming units (CFU) in all coatings for *E. coli*, CFU for *S. aureus* was reduced by adding 1.5 wt.% of Cu to the coating. The biocompatibility testing showed a cytotoxicity threshold at a Cu-release of 14.3 mg/L in 120 hours. Based on our findings, we suggest medium gas parameters for HVSFS and the addition of 1 wt.% Cu to the coating. With these parameters, a reasonable antibacterial effect can be achieved while maintaining sufficient biocompatibility.

**Graphical Abstract:**

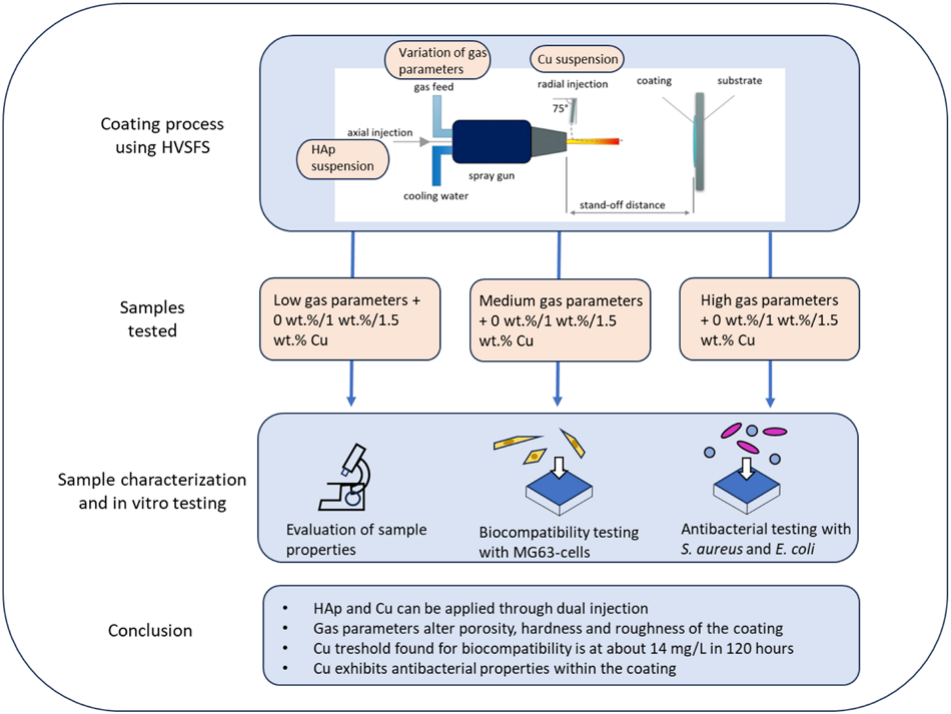

## Introduction

Implantation of an artificial joint (endoprosthesis) is a very effective form of therapy for the treatment of both degenerative joint diseases and traumatically induced lesions of the bone. Nevertheless, there are problems such as early loosening of artificial joints [1], the lack of stability of titanium implants in bone weakened by osteoporosis [2, 3] and implant-associated infections [4].

One way to improve integration into the bone is to coat metallic implant bodies with osteoconductive ceramic materials [5, 6]. The coating thickness is always a compromise between the solubility of the ceramic coating and its mechanical properties. A coating thickness greater than 100 µm increases the risk of fatigue fractures of the ceramic due to shear, bending, tensile, and compressive forces. Thick coatings also lead to increased delamination and fragmentation [7, 8]. The coating thickness should therefore be set in a range of 50–100 μm [9, 10]. Thinner coatings also generally have higher adhesive strengths compared to thicker coatings [8].

Since the layers are only needed to achieve a strong bone-to-implant contact, degradable coatings are to be favored [5, 11]. The primary goal of these materials is to minimize the time between primary stability (time immediately after implantation of the prosthesis) and the achievement of secondary stability (final anchorage in the bone). Degradation should be synchronized with new bone formation to ensure bone maturation and sufficient stability at the interface. The metallic base body can ensure this stability after degradation of the coating with sufficient roughness and surface structuring. This has been adequately demonstrated by numerous experimental and clinical data [12]. Coatings also offer the possibility of incorporating antibiotically active elements into the coating. In this way, antibiotic prophylaxis and accelerated osteointegration can be achieved simultaneously.

In previous work, we have shown that it is possible to produce thin films of resorbable bioactive ceramics such as hydroxyapatite (HAp) and bacteriostatic or bactericidal metals such as silver, copper, and bismuth using the high-velocity suspension flame spraying (HVSFS) process [13–15]. Due to the finer microstructure, the mechanical, physical, and biological properties of suspension-sprayed coatings differ significantly from those of thicker conventional coatings produced by atmospheric plasma spraying, despite having the same composition. The results of the in vivo studies show that the degradation of the ceramic coatings is predominantly solvent-based. For example, suspension-sprayed coatings exhibit significantly lower roughness and porosity values, which led to lower solubility of the coatings in the in vivo and in vitro studies, despite good biocompatibility [14]. The animal studies clearly showed incorporation of the coatings instead of degradation (publication in preparation). In addition, the biocompatibility tests [13] already showed that Bi is not suitable as an antimicrobial metal in the coating. Therefore, the current study was initiated to significantly increase the porosity using the HVSFS parameters.

The aim of the present study was to evaluate the technical properties, biocompatibility, and antimicrobial activity of coatings prepared by different gas parameters and Cu dotations.

## Materials and methods (Experimental procedure)

### Suspension

#### HAp suspension for axial injection

The axial suspension (SA) was used for axial injection. Raw HAp powder was ball-milled until a d90 value of 5 µm was reached. Then it was mixed with deionized water and 3 wt.% of a stabilizing agent based on phosphonate was added under continuous stirring using the Dissolver Dispermat® LC. The solid content was set to 5 wt.%.

#### Cu suspensions for radial injection

An aqueous radial suspension (SR) was prepared for the external radial injection. An electrolytic Cu Powder from GGP Metal Powder AG with a d90 value of 15 µm was used.

### Coating deposition

The coatings were deposited on two different substrates, i.e. titanium grade 2 specimen (ARA-T Advance GmbH, Dinslaken, Germany) and V2A stainless steel specimen (Schmiedekult, rapa GmbH, Emmerich am Rhein, Germany). The steel plates were used for technical characterization. Since the coatings will later be used on titanium implants, all biocompatibility and Cu release tests were performed on titanium.

Prior to coating, the samples were grit-blasted using F60 corundum at a pressure of 4 bar and subsequently cleaned with acetone in an ultrasonic bath. Finally, the samples were weighed to allow accessing the deposition efficiency.

**Fig. 1:**
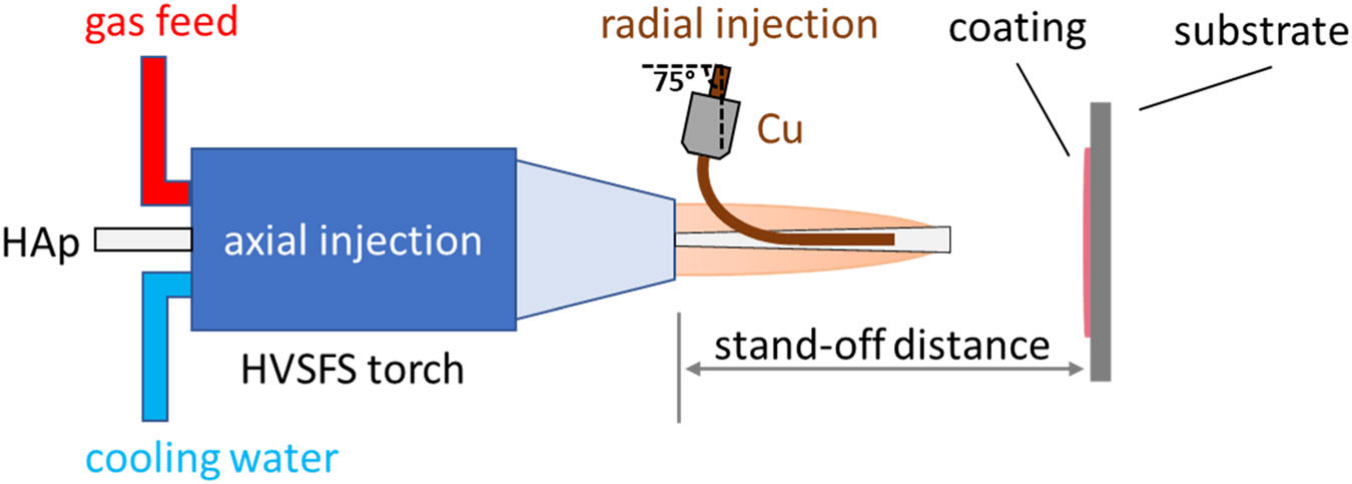
Schematic description of HVSFS process equipped with an axial and radial injection line. Radial injection is tilted towards upstream direction (75°) (based on our previous works [16, 17])

A modified TopGun-G system (GTV Verschleißschutz, Luckenbach, Germany) was used for this work. The torch was mounted on a six-axis robot to perform controlled meander movement with an offset of 3 mm and a spraying distance of 120 mm. Five meander cycles were performed in front of the substrate surface during the HVSFS deposition process (Fig. 1). The relative surface speed was set to 600 mm/s. For all sprayed samples a 22-8-135 combustion chamber was used. An axial pressurized air cooling with two nozzles at the left and right side of the torch axis was used. Table 1.

**Table 1:** Summary of formulation of aqueous (di-H_2_O) suspensions with additives used

Material	Type/Manufacturer		Suspension denotation	Used additives type:	[wt.%]	solid content [wt.%]
HAp		Budenheim	SA	Phosphonate based	3	5
Cu		GGP Metal Powder AG	SR	hydrocolloid	3	0.25

A detailed description of the axial/radial injection principle and the device set-up were reported in previous studies AlN/Flourescin [16, 17]. The axial feed rate was set to 80 ml/min, and the radial feed rate was varied to obtain Cu contents of 1%, 1.5%, and 2 wt.% in the final coatings. An overview of all the coating parameters that were varied can be found in Table 2. The coating denotation is defined as 0-L ≙ 0 wt.% Cu, low gas parameters; 1-L ≙ 1 wt.% Cu, low gas parameters, 1.5-L ≙ 1.5 wt.% Cu, low gas parameters, 0-M ≙ 0 wt.% Cu, medium gas parameters; 1-M ≙ 1 wt.% Cu, medium gas parameters, 1.5-M ≙ 1.5 wt.% Cu, medium gas parameters, 0-H ≙ 0 wt.% Cu, high gas parameters; 1-H ≙ 1 wt.% Cu, high gas parameters, 1.5-H ≙ 1.5 wt.% Cu, high gas parameters.

**Table 2:** Overview of the low (L), medium (M) and high (H) scoating parameters

Coating denotation	SA federate [ml/min]	RA federate [ml/min]	Total gas flow [slpm]	C_2_H_4_ [slpm]	O_2_ [slpm]
0-L	80	-	195	70	125
1-L		16			
1.5-L		24			
0-M	80	-	230	80	150
1-M		16			
1.5-M		24			
0-H	80	-	300	90	210
1-H		16			
1.5-H		24			

### Feedstock and coating characterization

The feedstock morphology was investigated using a scanning electron microscope (SEM) S-800 (Hitachi High-Technologies Corporation, Tokyo, Japan).

To analyze if the suspensions were eligible for spraying, the particle size distribution was evaluated by laser diffraction using a Mastersizer3000 (Malvern, UK), and rheological measurements were carried out with a modular compact rheometer MCR 302 (Anton Paar, Austria).

#### Deposition efficiency

The deposition efficiency (DE) was determined according to DIN EN ISO 17863:2004 to obtain a quantitative evaluation of the coating. The mass difference Δm between the sample before and after the coating process was calculated and then divided by the weight of the coating material fed into the torch while spraying the substrate area.

#### Hardness

The coatings’ Vickers hardness was measured on polished cross sections using a Fisherscope H100 (Helmut Fischer GmbH, Sindelfingen, Germany) hardness tester. HV 0.03 scale was used according to DIN EN ISO 14577 standard. This scale was selected because of the presence of Cu in the coating which is ductile. The measurement was force-regulated and the applied load of 294.199 mN was applied for 20 s with a load and release time of 5 s. For each sample, thirteen imprints were placed on the cross-section of the coating to provide a base for the average hardness and its standard deviation.

#### Roughness

Coating roughness values R_a_ and R_z_ were investigated by tactile measurement with Mahr Perthometer (Mahr, Esslingen, Germany). The measurement was performed with a length of 17.5 mm and 5 single measurements according to DIN EN ISO 3274. For each coating, the values and standard deviation were determined by taking the average values.

#### Optical microscopy

Coating microstructures were analyzed through an optical microscope MeF4M (Leica GmbH, Wetzlar, Germany) in bright field. Images were taken and analyzed by the software a4i analysis (A4I, London, Great Britain). Coating thicknesses were characterized according to DIN EN ISO 1463:2021-08 by measuring fifteen single coating thickness values and respectively calculating average value and standard deviation.

#### SEM/EDX

Further details of the microstructures were observed using a field-emission scanning electron microscope (SEM) S-800 (Hitachi High-Technologies Corporation, Tokyo, Japan). Cross-section samples were sputtered with carbon before SEM examination. The SEM images were also used to assess the coating porosity with image processing software (ImageJ 1.47 v) based on a contrast threshold.

#### XRD

The phase composition of the coatings was analyzed by X-ray diffraction (XRD: X’Pert PRO, PANAlytical, Almelo, The Netherlands) using Cu–Kα radiation (wavelength: 0.1540598 nm). The diffraction patterns were collected in the 20°–70° 2θ range (step size: 0.02°; scan rate: 5 s/step).

#### ICP-MS

The Cu content in the coatings was determined by inductively coupled plasma mass spectrometry (ICP-MS: iCAP RQ, Thermo Fisher Scientific, Germany). Scraped layers (3.0 mg) were first dissolved in 69% nitric acid (1 mL; Carl Roth, ROTIPURAN® Supra) before being diluted in extra-pure water (1:100). The Cu amounts were measured against standard solutions of 9.8 mg/L. The values represent an average of 3 measurements.

### In vitro characterization

#### Preparation of the samples

All tests for in vitro characterization were performed using coated titanium specimens. The 5 × 5 cm coated titanium plates were cut into 1 × 1 cm samples using the Struers Secotom-50 (Willich, Germany) at a feed speed of 0.040 mm/s and a rotation speed of 3000 rpm. The blade (Struers Diamond Cut-off Wheel M1D15, Ø 152 mm × 0.4 mm) was cooled with recirculating deionized (DI) water, which was frequently changed. Afterward, the samples were washed and disinfected by immersing the samples twice in 70% and once in 100% Ethanol for 5 min each. Furthermore, the samples were autoclaved in Systec D-Series Horizontal Benchtop autoclave (Thermo Fisher Scientific, Germany).

#### Eluent experiments

According to ISO standard 10993-15:2019-11, 3 samples of HAp coating (with/without Cu doping) were incubated in Thermo Scientific 15.0 mL tubes with 6 mL Tris buffer (TRIS hydrochloride, 1 kg Pufferan® ≥ 99%, p.a., Article No. 9090.3, Roth®, Karlsruhe, Germany) for 5 days at 37 °C. The pH of the Tris buffer was adjusted to 7.4 with hydrochloric acid solution (hydrochloric acid solution, volumetric, Reag. Ph. Eur., 0.1 M HCl (0.1 N), Honeywell, Charlotte, NC, USA). After 1, 2, 3, 4, and 5 days, the samples were transferred to a new 15 mL tube, 6 mL of Tris buffer was then added. The collected solutions per day and the samples were analyzed separately at the Institute of Geosciences, University of Freiburg, Germany, using an atomic absorption spectrometer (Perkin Elmer AAS 4110ZL Zeeman, Perkins Elmer, Waltham, MA, USA).

#### Biocompatibility testing

##### Cell culture

MG-63 cells (ATCC, CRL 1427) were used for all biocompatibility tests. The cells were maintained using a default medium consisting of Dulbeccos Modified Eagle Medium DMEM/F-12 (DMEM/F12) (Gibco, Braunschweig, Germany), 10% fetal bovine serum (Biochrom, Berlin, Germany) and 1% Penicillin/Streptomycin (Gibco, Braunschweig, Germany). The cells were passaged by Trypsin/Ethylene diamine tetracetic acid treatment twice a week. The medium was exchanged every other day. The cells were kept at 37 °C, 5% CO_2_, and 100% humidity.

All tests were performed with 50,000 cells/75 µL per sample. Per test, 3 identical samples were used and all tests were repeated at least 3 times.

##### Live/Dead assay

On each sample, 75 µL of the medium was pipetted with 50,000 cells/75 µL of MG-63. As a negative control (C-), the cells were also seeded onto a Thermanox ® cover slip. The well plates were then incubated for 2 h at 37 °C and at a CO_2_ saturation of 5% in an incubator. After two hours, 1 mL of the specific cell medium described previously was added to each well before incubating the well plates in the incubator for 1, 3, and 7 days. The staining solution was prepared by adding 2 mL DPBS (art. No. 14190-094, Gibco, Grand Island, NE, USA) to a Falcon tube (Greiner Bio-One International GmbH, Kremsmünster, Austria) and 4 µL ethidium homodimer III (Eth D-III) solution (together with the calcein part of the Live/Dead Cell Staining Kit II (PromoCell, Heidelberg, Germany)), according to the manufacturer’s protocol (PromoCell). Then, 1 µL of calcein dye (Calcein-AM) was added after mixing the staining solution. All steps were performed in the dark to avoid photobleaching of the staining solution and samples. To eliminate serum esterase activity, all samples at a specific time point had the medium removed and the cells washed. The samples were then immersed in the staining solution for 10 min. Afterwards, they were washed with DPBS. The evaluation was performed using an Olympus fluorescence microscope (BX51, Olympus, Osaka, Japan) at 4 different positions on the samples, 1 overview and 3 detailed images, at 5× and 10× magnification. Under the fluorescence microscope, living cells appear bright green. Calcein-AM is able to penetrate the cell membrane and is cleaved by esterases into green fluorescent Calcein. Eth D-III can penetrate through defective cell membranes, intercalate with DNA and emits red light. Dead cells therefore appear bright red. Cells that appeared in a faint red color were classified as unhealthy cells.

##### Cell proliferation (WST-I)

The cells were again seeded onto the different coatings (with/without Cu doping) and, as a negative control (C−), onto Thermanox® cover slips in the same numbers and concentrations as in the previous biocompatibility tests. After two hours of incubation in an incubator at 37 °C and 5% CO_2_ saturation, the cells adhered to the samples and Thermanox® cover slips. 1 mL of DMEM-F12 was added to all wells, with 1% P/S and 1% FBS added. Since FBS at higher concentrations can lead to background absorption, only 1% FBS was used. Further cell proliferation assay was performed according to the manufacturer’s protocol (see our other work [18]). Finally, 10% WST solution was added to all samples at the respective measurement time point (1, 3, 7 d) and incubated for 2 h in the incubator. After 2 h, absorbance was measured with a spectrometer (SpectroStar nano, BMG Labtech, Ortenberg, Germany) at 450 nm.

##### Cytotoxicity (LDH assay)

LDH measurements were performed after 24, 48 and 72 h. In addition to the samples, positive controls (Triton X, 100% toxicity, C+) and negative controls (cells only, 0% toxicity, C−) on a Thermanox® cover slip were used for the measurements at different time points. Both Cu-doped and Cu-free HAp coatings were also examined. Cells were seeded onto the scaffolds and membranes in 75 µL of their medium (MG-63: 50,000 cells/75 µL). These were then incubated for 2 h in an incubator at 37 °C and 5% CO_2_ saturation. Subsequently, 1 mL of DMEM-F12 medium was added to all wells, with 1% P/S and 1% FBS added. After 24 h of incubation, 100 µl from each well was transferred to three new wells of a 96-well plate. Thus, 3 wells containing 100 µl each were obtained from one well. To evaluate cytotoxicity, the cytotoxicity detection kit solution was prepared. For this purpose, 111.1 µL of the catalyst solution was mixed with 5 mL of staining solution. Of this, 100 µL was pipetted into each well before the plate was incubated in the dark for 30 min. At the end of the 30-minute incubation period, absorbance at 490 nm could be measured using a spectrometer (SpectroStar nano, BMG Labtech, Ortenberg, Germany). The experiment was repeated at least 3 times.

#### Antimicrobial testing

Safe Airborne Antibacterial Assay (SAAA) was performed to investigate the antimicrobial properties of the HAp coatings with and without Cu doping on *Staphylococcus aureus* (ATCC29593) and *Escherichia coli* (ATCC29522). The method was applied following the developers Al-Ahmad et al. and the exact experimental setup is described in detail elsewhere [19]. For this experiment, a suspension of 10^7^ germs was prepared in Tryptone Soya Broth (TSB) medium (Oxoid, Altrincham, UK). 1 mL of the suspension was mixed with 100 mL of sterile 0.9% NaCl solution (B. Braun, Melsungen, Germany) and placed in the glass bulb of the standard chromatography sprayer. Each sample was fixed on a Petri dish with double-sided tape and placed 15 cm away from the standard chromatography spray head. An air volume of a 60 cm^3^ syringe filled twice was used to spray the samples with the bacterial suspension. Petri dishes containing the samples were incubated for 30 min at 37 °C, 5% CO_2_ and 100% humidity. Then, the samples were wetted with 50 µL of 0.9% NaCl solution and incubated again for 2 min. The liquid obtained on the sample surface was applied to fresh Columbia agar plates containing 5% sheep blood (Oxoid, Altrincham, UK). After 12 to 24 h of incubation, the number of colony forming units (CFU) on the plates was counted.

#### HAp formation in simulated body fluid (SBF)

Simulated body fluid (SBF) was prepared as described by Jalota et al. [20]. The pH of the solution was adjusted to 7.4 with hydrochloric acid solution. A set of three samples was placed into a 24-well plate and each covered with 2 mL of SBF. After 14 days at 37 °C the samples were rinsed with double distilled water and left to dry. The HAp formation was evaluated electron-microscopically at Freiburg Materials Research Center, Freiburg, Germany. To confirm the formation of HAp, XRD measurements were performed.

### Statistics

Data were presented as mean ± standard deviation and analyzed by one-way analysis of variance (ANOVA). Comparison of mean values was performed according to Fisher LSD. The statistical significance level was set at *p* < 0.05. In the graphs, significant differences between different samples were marked with (*). Horizontal lines indicate between which samples the significant difference exists. Calculations were performed using OriginPro 2023 SR1 (OriginLabs, Northampton, MA, USA).

## Results

### Suspension results

**Fig. 2:**
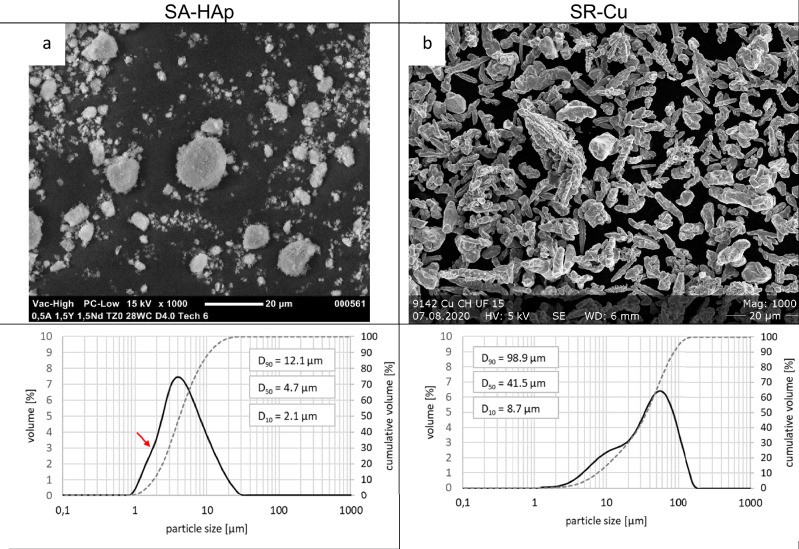
Particle size distribution of suspensions SA (**a**) and SR (**b**), with corresponding SEM images of raw powders

The particle size distributions of both suspensions can be seen in Fig. 2. SA-HAp shows a monomodal distribution with a d_50_ value of 4.7 µm. A minor shoulder on the left side of the distribution can be seen (red marker). The used raw powder has a d_50_ of 5 µm and, accordingly, the particles were well dispersed and stabilized. The SR-Cu suspension shows a bimodal particle size distribution. The metal particles could not be completed deagglomerated. The primary particle size of the Cu Particles (15 µm) builds the left shoulder. In general, the particle size distribution shows a d_50_ of 41.5 µm.

### Coatings results

#### Deposition efficiency

**Fig. 3:**
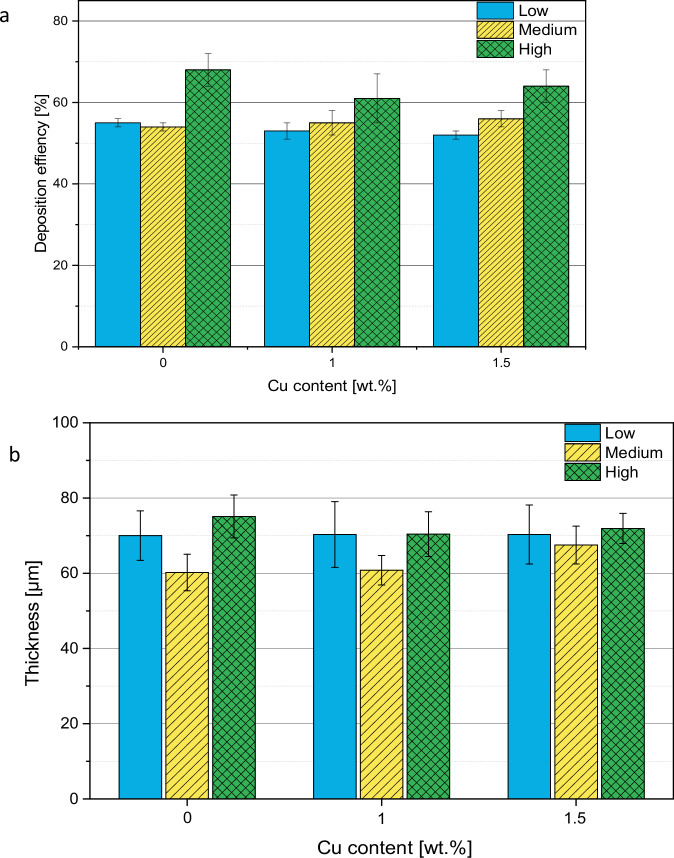
Deposition efficiency (**a**) and thicknesses (**b**) of coated samples

The deposition efficiencies (DE) and the coating thicknesses of all samples were determined. The DEs vary from 50% to 68%. There is no significant observable trend between the coatings that were coated with the low and medium gas parameters (Fig. 3a). For the high gas parameters, DEs are higher. The coating thickness is generally around 70 µm and shows the same trend but weaker (Fig. 3b).

#### Hardness and roughness

**Fig. 4:**
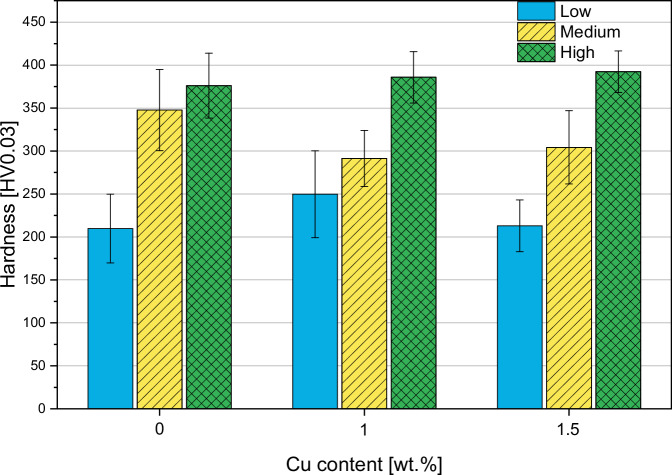
Hardness of coated samples

The hardness is strongly dependent on the gas parameters. As Fig. 4 shows, there is a significant hardness increase which is corresponding to the increase of total gas flow conditions (L < M < H). This is likely caused by the different porosity as shown in the next paragraph. The L parameters result in the lowest hardness results (Fig. 4). The Cu content does not influence the hardness significantly.

**Fig. 5:**
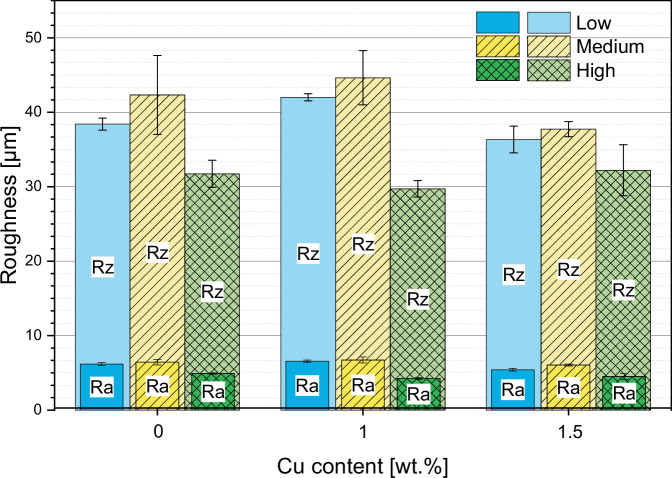
Roughness Ra and Rz of coated samples

The analysis of surface roughness under low and medium gas parameters revealed a minimal difference in roughness values (Fig. 5). This suggests that within this range of gas parameters, the impact on coating roughness is relatively low. Coatings subjected to high gas parameters exhibited a significant reduction in surface roughness. The results indicate that higher gas parameters contribute to well-molten splats which create a smoother and more uniform coating surface.

#### SEM/EDX

**Fig. 6:**
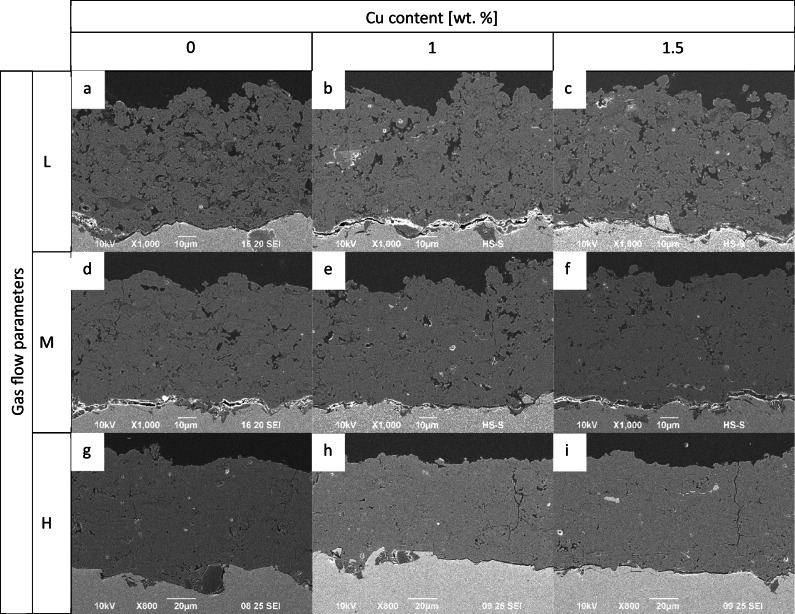
Coating microstructures investigated by SEM. It can be seen that porosity decreases with increasing gas flow parameters. Cu splats are more visible when Cu-content is increased

Scanning Electron Microscopy (SEM) was used to investigate the microstructure of coatings prepared under varying gas parameters and Cu content as shown in Fig. 6. Notably, porosity depends on these parameters, with higher gas flows resulting in lower porosity. Additionally, the SEM analysis unveiled the presence of cracks in coatings prepared under high gas parameters, indicating residual stress in the coating (Fig. 6g–i).

**Fig. 7:**
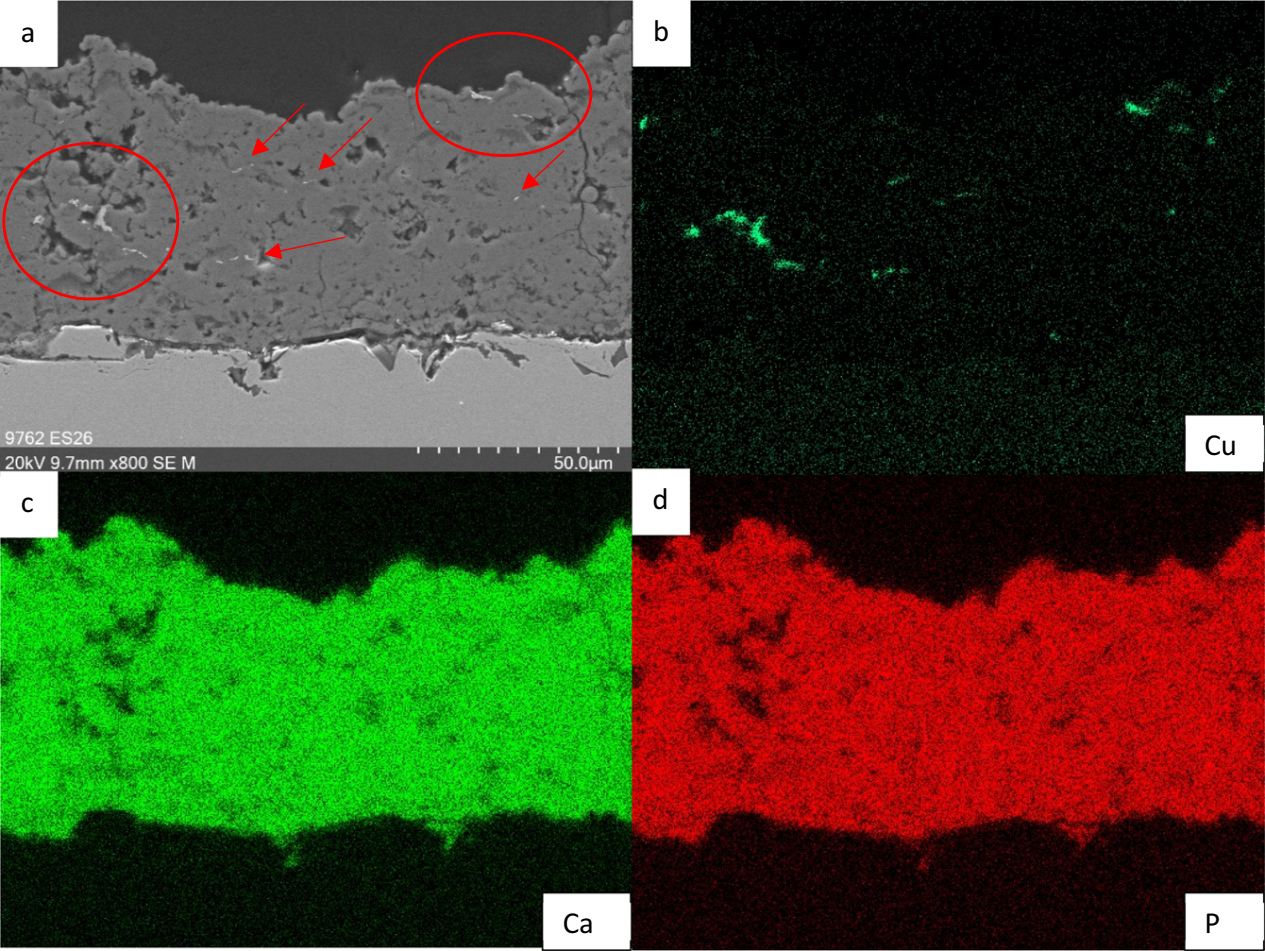
Example of EDX: Sample 1.5-M. It can be recognized that the brighter splats in the coating match with the EDX pattern of Cu. The rest of the coating is as expected made of Ca and P

Furthermore, Energy Dispersive X-ray Spectroscopy (EDX) was used to confirm the incorporation of Cu into the coatings (Fig. 7). Bright regions in the SEM images in Fig. 7a corresponded to a high Cu concentration as confirmed by EDX (Fig. 7b), validating the successful integration of this metal into the HAp matrix.

#### Porosity

**Fig. 8:**
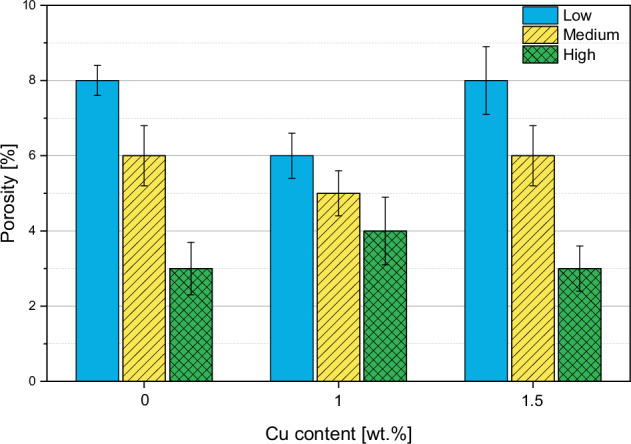
Porosity of all coated samples

As can be seen in Figs. 6 and 8, the porosity decreases with increasing gas flow. The coatings that are sprayed with the low gas parameters reach the highest porosity of about 8%. The H parameters result in the lowest porosity with a value of max. 4%.

#### XRD

**Fig. 9:**
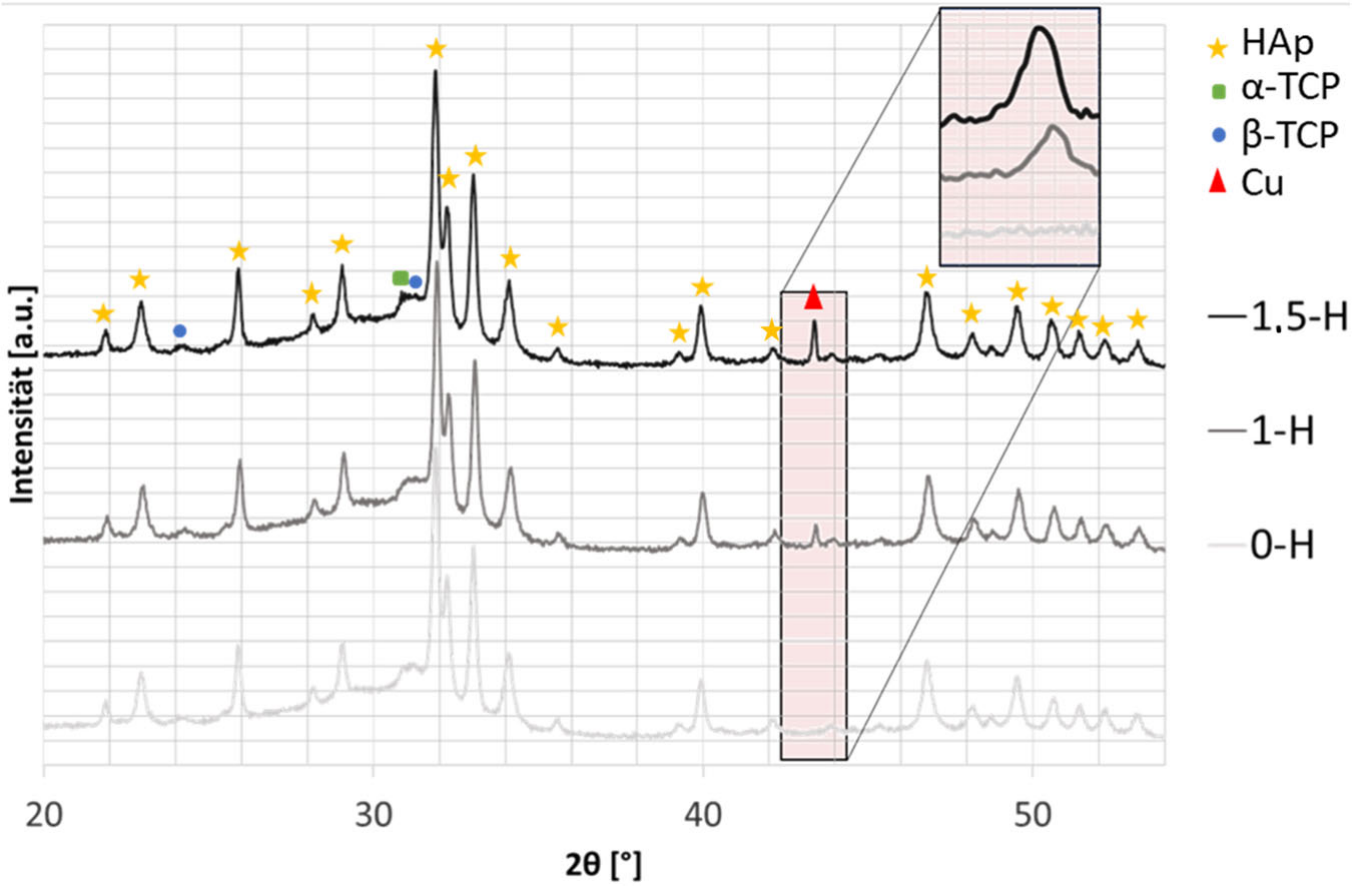
XRD patterns of the samples sprayed with high gas flow parameters. It is visible that the more Cu content was radially sprayed, the higher the Cu peak is. Besides HAp, there are also some minor TCP peaks due to the high temperatures which lead to HAp decomposition

X-ray Diffraction (XRD) analysis was used to evaluate the characteristics of the coatings, with a specific focus on the successful incorporation of Cu. As depicted in Fig. 9 XRD patterns of the samples sprayed with high gas flow parameters. It is visible that the more Cu content was radially sprayed, the higher the Cu peak is. Besides HAp, there are also some minor TCP peaks due to the high temperatures which lead to HAp decomposition, the XRD spectra showed a Cu peak that increased proportionally with higher Cu content in the coatings. The XRD patterns also revealed the dominance of HAp peaks, with some contributions from tricalcium phosphates (TCP).

#### ICP-MS

**Fig. 10:**
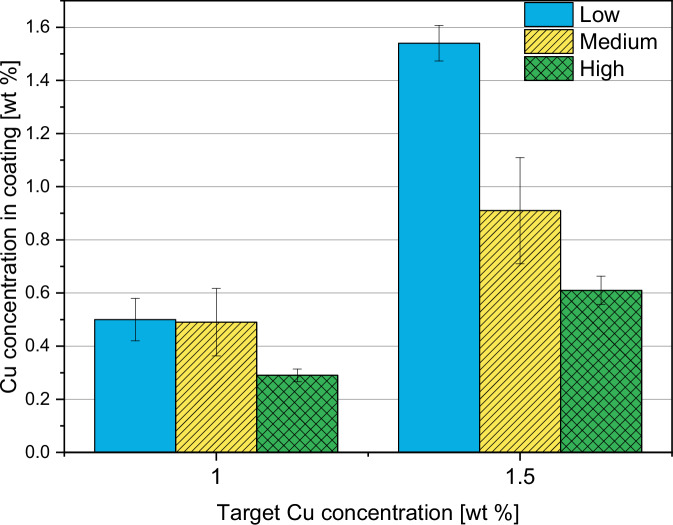
Cu concentrations in the coatings measured by ICP-MS

The results of the measured Cu amounts in the coatings by ICP-MS are shown in Fig. 10. The radial feed rate was adjusted during the coating process to achieve Cu concentrations of 1.0 wt.% or 1.5 wt.%. The target Cu concentration of 1.5 wt.% could only be achieved using coating parameters L. Deviations of 51–71% were observed for all other samples. The lower the gas flow parameters (L < M < H), the higher the obtained Cu concentration, and the Cu concentration in the coating was closer to the starting composition. As a result, more Cu is deposited on the layers by using lower gas flow parameters. In order to verify that the Cu-free reference samples (0-L, 0-M, 0-H) did not contain Cu, they were also measured by ICP-MS. In fact, only a negligible amount of Cu (<0.02 wt.%) was found, probably due to contamination during the measurements or during the coating process.

### In vitro testing

#### Eluent experiment

The Cu release of all samples containing Cu was between 9.54 and 18 mg/L. The exact values are shown in Table 3.

**Table 3:** Cumulative Cu release over 120 h [mg/L]

	24 h	48 h	72 h	96 h	120 h
0-L	0.13	0.18	0.21	0.24	0.27
1-L	10.71 ± 2.49	11.61 ± 0.1	12.09 ± 0.05	12.43 ± 0.03	12.73 ± 0.03
1.5-L	14.7 ± 1.3	16.11 ± 0.23	16.89 ± 0.1	17.49 ± 0.06	18 ± 0.07
0-M	0.21	0.26	0.3	0.34	0.38
1-M	8.23 ± 1.55	9.11 ± 0.13	9.47 ± 0.04	9.38 ± 0.04	9.98 ± 0.07
1.5-M	11.87 ± 1.51	13.01 ± 0.15	13.53 ± 0.09	13.91 ± 0.06	14.27 ± 0.07
0-H	0.33 ± 0.06	1.1 ± 0.06	2.1 ± 0	2.37 ± 0,06	2.97 ± 0
1-H	4.97 ± 0.85	6.54 ± 0.15	8.04 ± 0.10	8.67 ± 0.06	9.54 ± 0.06
1.5-H	5.60 ± 0.70	7.47 ± 0.15	8.14 ± 0.06	8.97 ± 0.06	10 ± 0.06

The highest Cu release could be detected within the first 24 h. After that, the Cu release gets significantly lower and approaches a plateau. In all groups, the Cu release is congruent with the amount of Cu in the coating. Coatings with a low Cu concentration release a small amount of Cu, while coatings with a high Cu concentration release a large amount of Cu. Comparing L, M and H at the same Cu concentration, it can be seen, that the Cu release decreases with higher gas flow parameters.

#### Biocompatibility

##### Live/Dead assay

**Fig. 11:**
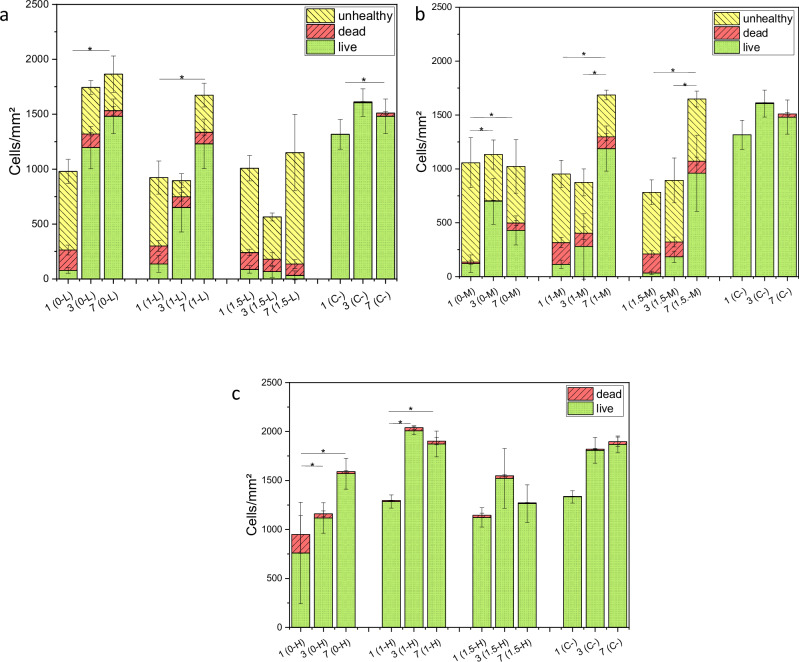
Live, dead and unhealthy cells after 1, 3, and 7 days for L (**a**), M (**b**) and H (**c**) with cells on Thermanox membrane as negative control (C−). *p* < 0.05 (*) for live cells only

As Fig. 11 shows, all low, medium and high gas flow groups show different live/dead cell counts and different proliferation kinetics over seven days.

For the low gas flow group, the sample 0-L and 1-L show a confluent cell layer after seven days with a comparable number of live cells as the control group. It is notable, that the cell count of dead and unhealthy cells is very high on the first day, but drops down to a noticeably lower count on day 3. The same tendencies can be observed for 1.5-L. The cell count of dead and unhealthy cells for 1.5-L then increases in between the third and seventh day, whereas almost no live cells can be found on those samples. The total cell count for 1.5-L is lower than for 0-L and 1-L.

For the medium gas flow group, again a high number of dead and unhealthy cells can be found on the first day, which then drops down on day 3 for 0-M and 1-M. Both 1-M and 1.5-M show confluent cell layers on day 7, but 1.5-M exhibiting a significant number of dead and unhealthy cells, too.0-M shows a lower cell count than 1-M and 1.5-M.

For the high gas flow group, no unhealthy cells can be detected. Also, the number of dead cells, except for 0-H on day 1, is lower compared to the low and medium gas flow group. In all samples, a confluent cell layer after 7 days can be found. For the samples containing Cu, the alive cell count decreases with higher Cu concentration. It is noticeable that the cell count decreases between day 3 and 7 in those samples.

##### Cell proliferation (WST-I)

**Fig. 12:**
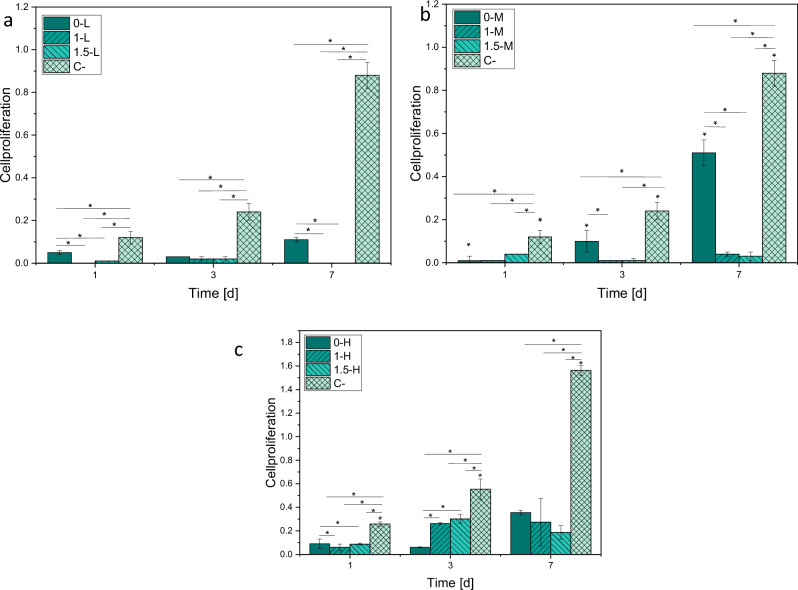
Cell proliferation on day 1, 3 and 7 for L (**a**), M (**b**) and H (**c**). Medium with cells serves as a negative control (C−). *p* < 0.05 (*)

Figure 12 shows that in comparison to the control group, almost all HAp samples show a significantly slower cell proliferation rate. Samples with HAp coating containing no Cu, show positive proliferation tendencies over 7 days in L, M and H. Cell proliferation is higher for samples with higher gas flow. Within the group of samples containing Cu, proliferation kinetics are similar to each other. In between day 1 and 3, slight cell proliferation can be detected, whereas between day 3 and 7 the proliferation subsides. The cell proliferation on samples containing Cu is lower compared to the HAp sample containing no Cu.

##### Cytotoxicity (LDH assay)

**Fig. 13:**
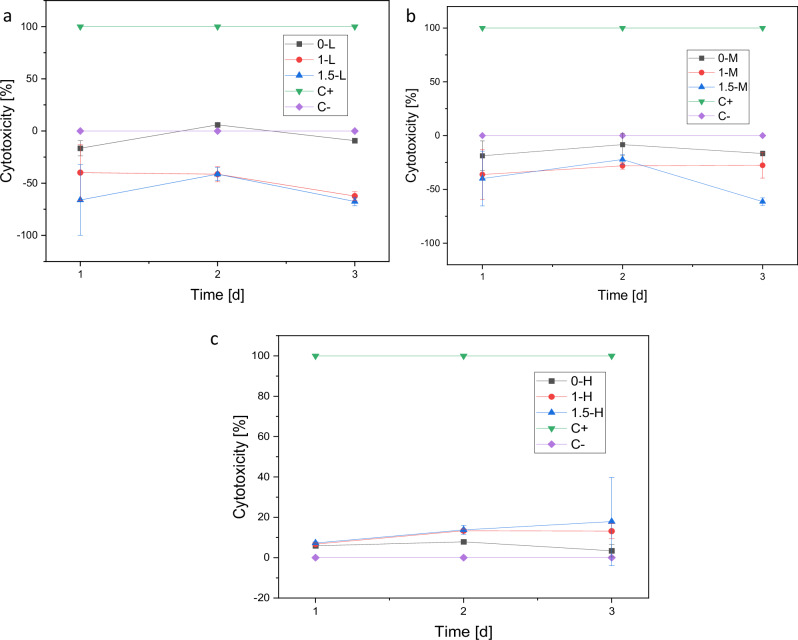
Cytotoxicity on day 1, 3 and 7 for L (**a**), M (**b**) and H (**c**). Triton X on cells serves as positive control (C+, 100% cytotoxicity), medium with cells serves as negative control (C−, 0% cytotoxicity). *p* < 0.05 (*)

As Fig. 13 shows, both low and medium gas flow groups show no cytotoxicity in comparison to the positive control. Only for 0-L slight cytotoxicity can be detected on day 2. On the third day, no cytotoxicity is shown for that sample. For the high gas flow group, cytotoxicity of 13 to 34% on day 3 can be detected. It is highest for the sample containing no Cu.

#### Antimicrobial testing

**Fig. 14:**
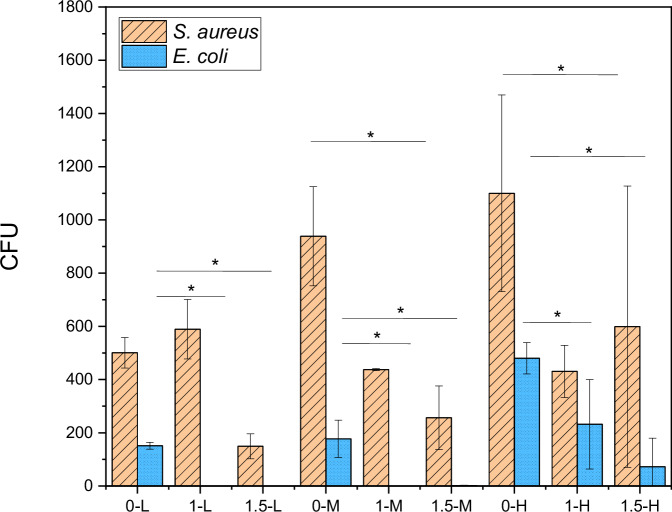
Number of Colony forming units per plate after a 30-minute incubation period

Figure 14 shows that for *E. coli*, all of the Cu-containing samples show very good antibacterial effects in comparison to the samples containing no Cu. With the presence of Cu, the number of colony forming units (CFU) can be reduced to 0 to 6 CFU per plate. Samples containing no Cu show a significantly lower number of CFU for *E. coli* than for *S. aureus*. For *S. aureus*, the formation of colonies can be reduced in all Cu-containing samples. The addition of 1 wt.% Cu can already reduce the number of CFU by almost half compared to the samples containing no Cu. By adding 1.5 wt.% Cu, another significant reduction of CFU can be achieved.

#### HAp formation in simulated body fluid (SBF)

**Fig. 15:**
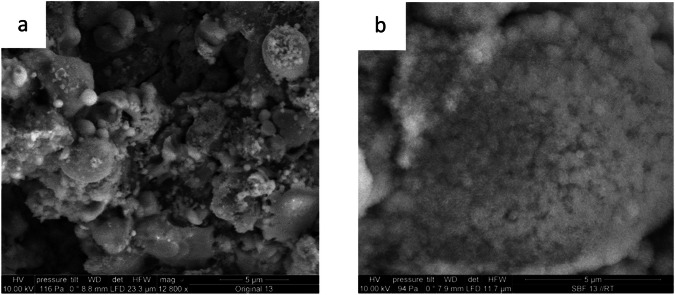
Before (**a**) and after (**b**) 14-day immersion in SBF

As can be seen in Fig. 15, structural changes can be detected in all samples after 14 days in SBF solution. SEM imaging shows the formation of crystalline structures, suggesting a new formation of HAp. XRD measurements confirm the formation of HAp. The density of those HAp crystals is similar for different gas flows.

## Discussion

### Suspensions

Both suspensions were stable and easy to process without any clogging. The Cu particles did not deagglomerate well, but this did not cause any issues during the coating process. The observed increase in particle size distribution in the Cu suspension compared to the powder may be due to a measurement artifact by gas bubbles. The high viscosity of the suspension is likely responsible for the formation and immobilisation of bubbles, which can range in size from 40 to 500 µm in water. These bubbles may be mistakenly detected as particles [21]. We did not observe any Cu agglomerates in the coatings, so we believe that the PSM results show this type of artefact. However, different amounts of Cu were measured, which is discussed in below with the ICP-MS results. Sedimentation of the particles could be avoided through the high viscosity of the suspension.

### Coatings

The deposition efficiency of the HAp coatings applied by thermal spraying is within the normal range or slightly higher than reported in literature [22]. By increasing the gas parameters, a slightly higher deposition efficiency can be achieved. The coating thickness varies between 60 and 70 µm, which is the target and is often found in literature [8–10, 23].

The HAp coatings produced with low gas parameters resulted in a hardness of 200–250 HV, which is relatively low. This is likely due to the high porosity of the coatings, as these values are consistent with literature data for coatings with similar porosities [24]. On the other hand, the coatings produced with medium and high parameters, which were less porous as well, exhibited significantly higher hardness values, ranging from 280–380 HV. These values fall within the usual range for HAp coatings applied by APS [25]. Thus, it appears that porosity and microhardness are inversely proportional: the less porous the coating, the harder it is. However, it is challenging to determine if this result is dependent on the measurement method, as it is difficult to ensure that only the coating material, and not the pores, is being measured. In fact, even though the points at which the micro-indenter takes measurements are manually selected to avoid pores, a more porous coating increases the likelihood that pores filled with embedding resin are measured instead of the actual coating material. This could result in artificially lower hardness values.

According to our experience, porosities ranging from 1% to 15% can be achieved through HVSFS, which is consistent with state-of-the-art APS HAp coatings that have a porosity of 5–15%. Dalton et al. (1995) conducted a comparison of commercially available HAp-coated implants and found that their porosity ranged from 5% to 14% [26]. It is generally recommended to aim for a porosity of below 9% for HAp coatings to avoid mechanical instability [27]. In this study, changing gas parameters, porosities of 4% to 8% could be achieved. That gas parameters can be adjusted to control the porosity was already described in literature [28]. Here, it could be observed that porosity decreases with increasing gas parameters. In fact, through higher gas parameters, higher particle velocities are achieved, resulting in higher flattening on the substrate and denser layers [29]. The study also revealed that Cu content did not have a measurable effect on porosity, which was expected given that Cu only makes up 1–1.5% of the coating. Lower gas flow parameters result in higher Cu concentration, with experimental values closer to the targeted amount of Cu. Higher gas flow rates result in increased kinetic energy of the gas flow, which may hinder the penetration of the radial Cu suspension into the flame jet compared to lower gas flow rates. This could account for the lower Cu content observed in coatings applied at higher gas flow rates. Although Cu splats were rare, they were evenly distributed throughout the cross-sections. EDX mapping could be used to detect higher Cu content.

Regarding the coatings’ microstructure, the cracks observed at high gas parameters are likely caused by residual stresses induced by the tensile quenching stresses in the coatings [30]. These stresses were previously reported and occurred as the layer cooled from solidification temperature to the equilibrium surface temperature [29]. Further investigations should analyze this in detail.

XRD analysis revealed the dominance of HAp peaks, with minor contributions of α- and β-Tricalciumphosphate (TCP). The plasma sprayed HAp coatings undergo dehydroxylation and thermal decomposition due to the extremely high temperatures in the plasma [31, 32].

This leads to the formation of TCP, tetracalcium phosphates (TTCP), and even cytotoxic calcium oxide (CaO) in the coating [32]. The coatings in this study do not exhibit any contributions from TTCP or CaO. By adjusting the gas parameters and increasing the suspension feed rate, the temperature experienced by the in-flight particles was kept lower than that of plasma-sprayed particles [33, 34].

These findings are consistent with our previous experiments [35, 36]. The XRD spectra demonstrate the successful introduction of Cu in the coatings without inducing structural alterations in the host material.

### Eluent experiment

For samples containing no Cu, traces of Cu could be detected in the eluates. This most likely can be explained by contamination during the coating process or while cutting the samples.

The release test shows a decrease in Cu release with higher gas flow parameters. It can be assumed that there is a relationship between porosity, hardness, and solubility of the coating. Higher gas parameters lead to greater hardness and decreasing porosity, therefore resulting in decreased degradation and Cu release.

On the other hand, lower gas flow parameters lead to higher porosity, which decreases the mechanical integrity of the coatings [37]. If the mechanical integrity of the coating decreases, liquid can more easily permeate the coating which results in a better dissolution with a higher Cu release. It is described that not only general porosity affects the dissolution rate of the coating but also pore size [38]. Pore sizes were not further investigated in this study. The hardness of the coatings also affects the degradation. Mishra et al. investigated the correlation between hardness and solubility. They also found an inverse correlation between hardness and solubility, since the solubility depends on how easy it is to break intermolecular interactions. Those are stronger for harder materials [39]. With regard to this explanation, we had expected a lower Cu release for the high gas flow group. We suspect that the higher deposition efficacy for this group leads to a slightly thicker coating with more Cu compared to the other two groups. For this reason, more Cu could have been released.

### Biocompatibility

The live/dead assay shows differences in biocompatibility of the coatings depending on the Cu concentration within the coating. Results also differ between different gas parameter groups.

The occurrence of unhealthy and dead cells on day 1, which exceeds the amount of living cells, could be found in all samples of L and M. This might be due to subcultivation and seeding onto the new samples surface which can cause physical stress on the cells. It is described, that enzymatic dispersion, which is the underlying principle of subcultivation and seeding, causes stress and damage on vertebrate cells. Among others, this stress leads to abnormal structures of the cells, decline of cell growth rate and low seeding densities. This stress reaction can also be evoked by subculturing under suboptimal conditions, for example pH-change or temperature change [40]. Furthermore, it could be a foreign body reaction, which also occurs with other biocompatible osteosynthesis materials as has been described in other studies [41, 42]. Within the first 3 days of incubation on the samples, the number of dead and unhealthy cells decreases while the count of viable cells increases in samples containing no to 1 wt.% Cu, which supports the assumption of an initial stress reaction due to the seeding process onto the new surface. Hap coatings have been thoroughly investigated within the last few decades and are one of the most attractive biomaterials at present due to their biocompatibility, osteoconductivity, and similarity to bone [43]. Similar results regarding the biocompatibility could be found in our experimental setup. 0-L, 1-L, and 1-M show the formation of a confluent cell layer within 3 to 7 days of incubation. Cell count of the viable cells is comparable to the viable cell count of the control. At all times, except for day 1, the cell count of dead and unhealthy cells never exceeds the amount of living cells. Surprisingly, 0-M shows a low viable cell count and a relatively high number of dead and unhealthy cells. Due to the results of 0-L and the generally known biocompatibility of Hap, a higher count of live cells was expected. Possible reason for that finding might be an excessive cell growth for the size of the surface they were seeded on. When cells reach their maximum density, cell proliferation will reduce and cells might die. Another reason might be, that there weren’t enough nutrients in the medium for the amount of cells [44, 45].

Biocompatibility drastically decreases for 1.5-L. Depending on the gas flow, release of Cu and therefore biocompatibility differs. For 1.5-L and 1.5-M there is a difference of 3.73 mg/L in the Cu-release, 1.5-L releasing more Cu. This might explain the differences in viable cell count for those two samples. On day 7, almost no viable cells could be found on 1.5-L, whereas the cell count of unhealthy cells and dead cells was very high. For 1.5-M the cell count was significantly higher on day 7 compared to 1.5-L and only slightly lower compared to 1-M. The live/dead results show that biocompatibility depends on the release of Cu. In our experimental setup, using MG-63 cells, a total Cu release of over 14.3 mg/L within 5 days drastically reduces the biocompatibility of the HAp-Cu coatings. The literature describes different limits for the amount of Cu in biomedical applications. Jastrzębski et al. [46] found an amount of 2.76 at.% induces cytotoxicity of MC3T3-E1 cell line. Others report that 0.8 wt.% Cu does not affect biocompatibility but 2.0 wt.% is detrimental for bone regeneration. In this study, Cu release within the first 5 days was around 2.5 ppm for 0.8 wt.% CuO and 8 ppm for 2 wt.%, MC3T3-E1 cells were used to assess biocompatibility [47]. As the toxic copper concentration varies with different cell types, an exact limit for Cu cannot be specified. For the high gas flow group, copper release within 5 days was maximum 10 mg/L- live/dead staining shows a good biocompatibility of all samples. Surprisingly, the cell count was lower for 0-H than for 1-H. Reasons for that might be again a lack of nutrients. There is also the possibility that fewer cells were seeded onto the sample at the beginning of the experiment.

Another factor which contributes to cell adhesion and biocompatibility is the surface roughness. Generally, cells adhere better on rougher surfaces [48]. Since the surface roughness is quite similar for all coatings it can be assumed that the Cu concentration in the coating is the deciding aspect for the biocompatibility.

Whereas the live/dead assay clearly shows cytotoxic effects for samples with high Cu content in the low and medium group, LDH testing states no cytotoxicity for those coatings. Almost all values in the LDH assay are negative which indicates no cytotoxicity in relation to the positive and negative control. It could be possible that cell proliferation was very high on the negative controls, so that after reaching the maximum value, cell proliferation decreased and cells died due to lack of nutrients in the medium. In contrast, cell proliferation on the sample coatings could have been lower, so that the nutrients in the medium were sufficient and cells therefore survived and no LDH was released. Due to the underlying principle of the experiment, supplementary feeding of medium was not possible. Looking at the high gas flow group, cytotoxicity could be found in all samples. Interestingly enough, the cytotoxicity was highest for the sample containing no Cu. This aligns with the number of dead cells found on day 1 for 0-H in the Live/Dead assay. Still, those results are difficult to explain with regard to the Live/Dead and LDH results of the other two groups. Possibly the renunciation of the additional feeding, led to the measurable cytotoxicity. Cu interference with the LDH assay could also explain the divergent results. Several studies have shown, that LDH assay is not suitable for evaluating the toxicity of Cu-containing coatings [49, 50]. Cu inactivates LDH and therefore interferes with the results of LDH testing. Generally, MTT assay is another possible method to evaluate the cytotoxicity. However, it has been found that Cu interferes with the MTT assay, too [51].

The WST-1 assay shows the highest viability values for the samples containing no CU. While the live/dead assay often shows cell proliferation comparable to the control group for samples with low Cu content, the values in the WST assay for Cu containing samples are very low. This discrepancy might be due to interaction of Cu with the WST-1- kit. It has been found that CuO and CuCl_2_ interact with the WST-8 kit. The underlying principle to the WST-8 kit is similar to the WST-1 kit used in our experiment. The tetrazonium salt WST-8 is reduced to formazan by dehydrogenases that are expressed in viable cells. Cu ions interfere with the reduction step [52].

### HAp formation in SBF

Investigation of HAp formation in Simulated Body Fluid (SBF) was performed to determine the bioactivity of the coatings. Kokubo et al. [53]. found that investigating the HAp formation in SBF can make predictions about the in vivo bioconductivity of the material. The formation of an apatite layer favors adhesion of osteoblasts on the implant surface and allows for good bonding between bone and implant [54]. As described in several other studies [13, 43], HAp coatings have great bioconductive properties. Our SBF testing shows similar results: All of the coatings exhibit a bone-like apatite layer after immersion in SBF for 14 days. Incorporating Cu into the HAp coating does not change the bioconductivity of the coatings. Unabia et al. [55]. stated similar results. In their experiment, only higher Cu concentrations (10%) altered the bioconductivity of the coatings. It should be emphasized that there are limiting factors that restrict the transfer of the in vitro results to living organisms: Although the ion concentration of the SBF solution is similar to the concentrations in the blood, factors like serum proteins, that can alter the solubility of the implant coating or the influence of the musculoskeletal system on apatite formation are neglected in this experiment [56].

### Antibacterial effect

Periprosthetic infections occur due to biofilm formation. Within hours of bacterial adhesion, an immature initial biofilm forms. Further maturation of the biofilm takes place within 3 to 4 weeks [57]. At this point, treatment of the infection generally is only possible with removal and later exchange of the prosthesis [58] which is a burden for the patient and for the healthcare system. In mature biofilms, microorganisms are remarkably resistant towards antibiotics as compared to their planktonic counterparts [59]. To prevent periprosthetic infection and the following treatment, biofilm formation must be suppressed, which can be achieved by reduction of bacteria concentration on the implant surface during the initial microbial adhesion. One downside of exclusive use of HAp for implant coatings is its poor antibacterial property [43]. The addition of Cu can improve this aspect.

The experimental setup to investigate the antibacterial effect of our coatings shows a significant reduction for both gram-positive (*S. aureus*) and gram-negative strains (*E. coli*), which are the most common cause for acute periprosthetic infections [60]. Differences of the structure of the outer membrane of gram-positive and gram-negative bacteria explain the better antibacterial effect of Cu on *E. coli*. Due to lipopolysaccharides on gram-negative cell membranes, which are negatively charged upon physiological pH, there is a better interaction between Cu and the bacteria, allowing for a better antibacterial effect [61]. But still, the achieved reduction of *S. aureus* might be enough to prevent the initial microbial adhesion and subsequently the formation of health-threatening biofilms on prosthesis. Generally, bactericidal activity is defined as 99.9% reduction of bacteria [62]. For prevention of periprosthetic infections the required bacteria reduction is not exactly known [43]. Li et al. [63]. found a copper threshold at 37 µM by testing the growth inhibition on *S. aureus* und *E. coli*. Surprisingly, CFU for *S. aureus* is much lower for 0-L than for 0-M or 0-H. This might be due to the experimental setup. During incubation the samples run the risk of drying out depending on how much liquid reached the surface during the spraying process. If the bacteria dry out, fewer colonies can grow. The used airborne assay has been shown to be a realistic procedure to simulate material surface contamination in surgery [19]. Hence, the results of the present study indicate that the addition of Cu to the HAp coating has a preventive effect against initial microbial adhesion and thus against biomaterial-associated infections. A question that could not be answered with our experimental setups is the durability of the antibacterial effect. Furthermore, the setup only depicts the reduction of viable bacteria within 30 min of incubation on the coating. Nevertheless, it could be expected that the effect of Cu after more than 30 min may be higher due to the additional adhesion forces of the material surface causing a stress deactivation of cell membranes and increasing the dead adherent bacteria [64].

## Conclusion

This study evaluates the effects of different gas parameters in the HVSFS-process on the coating properties as well as the biocompatibility and antibacterial effect of Cu-containing HAp-coatings.

We demonstrated the feasibility of producing composite coatings using materials with different properties through the HVSFS process. This achievement was facilitated by using dual injection. HAp served as the matrix material and was sprayed axially, while Cu was injected radially into the hot flame. This radial injection method minimized thermal stress on Cu, consequently reducing its oxidation during the process.

It was observed that gas parameters have an impact on the deposition efficiency of Cu. When using low gas parameters more Cu is deposited into the coating than when using high gas parameters. Another aspect, that is influenced by the gas parameters are porosity and hardness. Low gas parameters lead to higher porosity and lower hardness than high gas parameters. Considering all of the biocompatibility tests (WST-1-Assay, LDH-Assay, Live/Dead staining) the coatings produced with high gas parameters show the best biocompatibility while it decreases for the medium and low gas parameter group. The addition of 1 wt.% metallic Cu to HAp coatings can already lead to a great reduction of *S. aureus* and *E. coli*. This can especially be seen for the low group and medium group. Here, the addition of Cu eradicated *E. coli* and reduced the CFU for *S. aureus* by at least half.

In conclusion, HVSFS enables us to produce thin coatings with controllable coating properties, e.g. porosity and antibacterial properties while still maintaining biocompatibility. Further research will focus on how to achieve higher antibacterial efficacy without compromising biocompatibility. Possible starting points would be the implementation of different forms of Cu or the successful introduction of an antibiotically effective substance into the coating.

## Data Availability

The data provided in this research is accessible upon request from the corresponding author.
